# Defibrotide for prophylaxis of sinusoidal obstruction syndrome/veno-occlusive disease (SOS/VOD) in pediatric high-risk patients: consensus guidelines from the European Society for Blood and Marrow Transplantation (EBMT)

**DOI:** 10.1038/s41409-025-02793-x

**Published:** 2026-01-13

**Authors:** Selim Corbacioglu, Rajinder Bajwa, Ali Bülent Antmen, Adriana Balduzzi, Jaap Jan Boelens, Francesca Bonifazi, Simone Cesaro, Fabio Ciceri, Antonio Colecchia, Fiona Dignan, Katharina Kleinschmidt, Kris M. Mahadeo, Antonio Pagliuca, Petr Sedlacek, Peter J. Shaw, Jerry Stein, Zofia Szmit, Francesco Tambaro, Elif Ince, Marta Verna, Akif Yesilipek, Marco Zecca, Paul G. Richardson, Mohamad Mohty, Krzysztof Kalwak

**Affiliations:** 1 Department of Pediatric Hematology, Oncology and Stem Cell Transplantation, University of Regensburg, Regensburg, Germany; 2 Division of Hematology/Oncology/BMT, Nationwide Children’s Hospital, Columbus, OH USA; 3 Pediatric Hematology and Stem Cell Transplantation Unit, Acıbadem Adana Hospital, Adana, Turkey; 4 Hematopoietic Stem Cell Transplant Unit, Department of Pediatrics, Fondazione IRCCS San Gerardo dei Tintori, Monza, Italy; 5 School of Medicine and Surgery, University of Milano-Bicocca, Monza, Italy; 6 Department of Pediatrics, Transplantation and Cellular Therapies Service, Memorial Sloan Kettering Cancer Center, New York, NY USA; 7 IRCCS Azienda Ospedaliera-Universitaria di Bologna, Bologna, Italy; 8 Pediatric Hematology Oncology, Department of the Mother and Child, Azienda Ospedaliera Universitaria Integrata Verona, Verona, Italy; 9 Hematology and Bone Marrow Transplantation Unit, IRCCS Ospedale San Raffaele, University Vita-Salute, Milano, Italy; 10 Gastroenterology Unit, CHIMOMO Department, University of Modena and Reggio Emilia, Modena, Italy; 11 Manchester University NHS Foundation Trust, Manchester, UK; 12 Pediatric Transplant and Cellular Therapy, Duke University, Durham, NC USA; 13 Department of Hematological Medicine, King’s College Hospital & Kings College London, London, UK; 14 Department of Pediatric Hematology and Oncology Teaching Hospital Motol, 2nd Medical School, Charles University Motol, Prague, Czechia; 15 Blood Transplant and Cell Therapies Program, The Children’s Hospital, Westmead, NSW Australia; 16 Schneider Children’s Medical Center of Israel and Sackler Faculty of Medicine, University of Tel Aviv, Tel Aviv, Israel; 17 Clinical Department of Pediatric BMT, Oncology and Hematology, Wroclaw Medical University, Wroclaw, Poland; 18 Stem Cell Transplantation and Cell Therapy Division—AORN Santobono Pausilipon, Napoli, Italy; 19 Division of Pediatric Hematology Oncology and Stem Cell Transplantation, Ankara University Faculty of Medicine, Ankara, Turkey; 20 Pediatric Hematology and Stem Cell Transplantation Unit, Medicalpark Antalya Hospital, Antalya, Turkey; 21 Fondazione IRCCS Policlinico San Matteo Pediatric Hematology-Oncology, Pavia, Italy; 22 Department of Medical Oncology, Dana-Farber Cancer Institute, Jerome Lipper Center for Multiple Myeloma Research, Harvard Medical School, Boston, MA USA; 23 Hematology Department, AP-HP, Hôpital Saint-Antoine, Sorbonne Université, Paris, France

**Keywords:** Intestinal diseases, Diseases, Gastrointestinal diseases

## Abstract

SOS/VOD is a life-threatening complication of hematopoietic stem cell transplantation, especially in children, with incidences reaching up to 15–20%. Despite efforts, SOS/VOD remains unpredictable with significant morbidity and mortality. High-risk criteria are clearly defined, and the pediatric EBMT diagnostic criteria have improved sensitivity, reducing treatment delays and enhancing outcomes. A meta-analysis combining retrospective and prospective studies found a risk ratio of 0.30 for SOS/VOD with defibrotide (DF) prophylaxis. Additionally, two prospective trials were conducted: the pediatric prevention trial (NCT00272948) and the Harmony Trial (NCT02851407), involving adults and children, with primary outcomes of incidence and SOS/VOD-free survival, respectively. The trials produced conflicting results regarding the effectiveness of prophylactic DF. Despite significant limitations of the Harmony trial, a direct healthcare professional communication (DHPC) from the European Medicines Agency (EMA) advised against prophylactic DF. This recommendation has serious consequences for children, especially infants, who are among the most vulnerable groups receiving HSCT. Therefore, a panel of experts issued guidelines for children at high risk for SOS/VOD, in which DF prophylaxis is considered justified. These guidelines include a weighted scoring system based on all relevant high-risk criteria to predict SOS/VOD, supporting decisions regarding the use of prophylactic DF in children.

## Introduction

Sinusoidal Obstruction Syndrome, formerly known as veno-occlusive disease (SOS/VOD), remains one of the most challenging early post-transplant complications. Characterized by destruction of hepatic venular and sinusoidal structures, SOS/VOD results in hepatic congestion, liver failure, and, in its severe form, multi-organ failure (MOF). SOS/VOD is increasingly reported during standard chemotherapeutic regimens in pediatric oncology [1, 2], the adaptation of post-transplant cyclophosphamide as graft-versus-host-disease prophylaxis (GVHD) [3, 4], and, most recently, in novel transplant-related contexts such as gene therapy/gene editing [5, 6].

Substantial differences between adults and children are well-established [7]. The average incidence of SOS/VOD in children has consistently been above 15% to 20% over the decades, with historically reported rates as high as 60%, compared to current incidences of less than 10% in adults [8, 9]. Anicteric SOS/VOD, which raises concerns about the requirement of hyperbilirubinemia (>2 mg/dL) as a diagnostic marker, occurs in up to 30% of patients. These differences (summarized in Table 1, adapted from Corbacioglu et al. BMT 2018 [7]) highlight the unique challenges of diagnosing and managing SOS/VOD in the pediatric population. Therefore, SOS/VOD remains a life-threatening complication of hematopoietic stem cell transplantation (HSCT), particularly in children.

**Table 1:** Differences between adults and children.

Criteria	Children	Adults
Incidence	• Approximately 20% • Up to 60% in high-risk patients	• Approximately 10%
Risk factors	• Additional paed risk factors • Infants • Paed/genetic diseases with incidences above average	• Established risk factors
Clinical presentation	• Late-onset hepatic VOD in 20% • Anicteric hepatic VOD in 30% • Hyperbilirubinaemia, if present —Is frequently pre-existent —Occurs late during hepatic VOD —Is typical of severe hepatic VOD	• Late-onset hepatic VOD is rare • Anicteric hepatic VOD is rare
Need for proper assessment of ascites and hepatomegaly	• High incidence of disease-related hepatomegaly and ascites pre-HCT	–
Treatment	• Defibrotide for severe hepatic VOD with MOD/MOF was associated with better results in children compared with adults	

Adapted from Corbacioglu S et al. *Bone Marrow Transplant*. 2018;138–145.

*MOD* multi-organ dysfunction, *MOF* multi-organ failure.

## Pathophysiology

The pathophysiology of SOS/VOD is complex, initiated at the sinusoidal structure of the liver. In brief, a triggered inflammatory dysregulation at the endothelial level activates adhesion molecules and, subsequently, the coagulation cascade. Increasing platelet consumption is the earliest clinical sign of this process. This self-sustaining cascade is exacerbated by expanding endothelial damage and sinusoidal permeability, leading to the deposition of fibrin and extracellular matrix in the space of Dissé. Subsequent vasoconstriction and fibrosis further impair hepatic blood flow, leading eventually to hepatocytic damage and subsequent MOF.

In summary, sinusoidal injury impairs hepatic venous flow with portal hypertension, and consecutive hepatocyte death is the hallmark of severe clinical complications of VOD/SOS, which in turn lead to multi-organ dysfunction [10].

## Risk factors

Recent advances in understanding the underlying mechanisms of SOS/VOD have led to the identification of both modifiable and unmodifiable risk factors [11], such as pre-existing liver disease, conditioning regimens, and genetic predisposition.

## Clinical presentation and severity

Clinically, SOS/VOD can present as often painful hepatomegaly, frequently accompanied by jaundice, fluid retention, and ascites, signaled by transfusion-refractory thrombocytopenia (tRT). Its severity ranges from mild dysfunction to fulminant liver failure. Although early diagnosis and prompt management can improve outcomes, the condition remains difficult to diagnose due to a nonspecific and variable range of symptoms, inconsistent clinical diagnostic criteria with differing sensitivities and specificities, and unpredictable disease progression. These developments have led to increased diagnostic awareness, preventive measures, and new treatment options, including defibrotide (DF), which has shown promise in reducing endothelial damage and significantly lowering fatality [12].

## Diagnostic criteria and their impact on incidence and severity

It must be acknowledged that the diagnostic criteria used significantly influence the incidence, morbidity, and mortality of SOS/VOD. The Seattle criteria, introduced first, incorporated clinical observations such as elevated serum bilirubin, painful hepatomegaly, and weight gain [13]. These were subsequently followed by the Baltimore criteria [14], which introduced as a major change the requirement of a defined bilirubin threshold (≥2 mg/dL)—the only reproducible laboratory parameter, therefore valued by the community—aiming to improve diagnostic specificity. However, requiring hyperbilirubinemia greatly affected the reported incidence and morbidity of SOS/VOD. Several trials showed that the incidence of SOS/VOD was at least doubled and even quadrupled when diagnosed according to Seattle criteria compared to Baltimore criteria [8, 15, 16]. A *post hoc* analysis of the T-IND trial (NCT00628498), which enrolled 803 patients with SOS/VOD, revealed an overall incidence of 23% for SOS/VOD diagnosis without elevated bilirubin, with 29% reported in children and 15% in adults. Eighty percent of children and half of the adult patients presented with anicteric SOS/VOD within the 21-day limit of the Baltimore criteria and would have been missed. Furthermore, the trial demonstrated that a larger proportion of patients with hyperbilirubinemia, in both adults and children, presented with severe SOS/VOD and MOF. However, the Kaplan-Meier-estimated day 100 survival was also high in patients who presented with anicteric SOS/VOD [17], indicating that bilirubin level is not a predictive marker for severity.

The high prevalence of SOS/VOD among children, combined with numerous unmodifiable risk factors and a high rate of anicteric SOS/VOD, along with other considerations, prompted the European Bone Marrow Transplant Society (EBMT) to establish pediatric-specific diagnostic criteria (pEBMT criteria) in 2018. The goal was to create sensitive criteria that trigger therapeutic intervention regardless of the onset of hyperbilirubinemia to reduce morbidity and mortality [18].

Consumptive tRT was first described by McDonald et al. [19] in 1993 as an early sign, while prolonged tRT was indicative of severe SOS/VOD. Since its publication in 2018, several studies have confirmed that the pEBMT criteria meet the requirements as an early and sensitive tool for anticipating the diagnosis of SOS/VOD in children, compared to the classical Seattle criteria [20–24].

In this context, the introduction of tRT serves as a sensitive and innovative marker for early detection of platelet consumption following a systemic sinusoidal inflammatory response. While the 1-h corrected count increment is used as the objective measure for alloimmunization post-transplant [25], SOS/VOD-related tRT, as implemented in the pEBMT criteria, is more a marker of consumptive platelet use that requires increased frequency of platelet transfusions despite an adequate 1-h increment.

## Defibrotide prophylaxis evidence

The issue of prophylaxis for SOS/VOD arose when it became clear that DF was an effective treatment for SOS/VOD, allowing children to benefit from preemptive/prophylactic intervention with DF [26–29]. Since DF primarily targets the endothelium, concerns emerged that symptoms such as ascites, hepatomegaly, and hyperbilirubinemia, caused by significant hepatic sinusoidal damage, might serve as late indicators for intervention.

Several retrospective and prospective studies summarized in a meta-analysis identified a risk ratio of 0.30 (95% confidence interval 0.12–0.71; *p* = 0.006) for developing SOS/VOD with DF prophylaxis compared to controls [30]. The DF prophylaxis questions were also evaluated prospectively in two pivotal trials with conflicting outcomes. The first trial was the pediatric prevention trial (NCT00272948), conducted with pediatric high-risk patients from 2006 to 2009. The primary outcome was the incidence of SOS/VOD by day 30 post-HSCT. Assuming a 25% incidence, the sample size was initially calculated as 290 patients and later adjusted to 360 patients due to an observed incidence of 20% in the control group based on the Seattle criteria. The trial demonstrated a significant reduction in SOS/VOD incidence from 20 to 12% (*p* = 0.05), along with decreased SOS/VOD-related morbidity, including renal failure (*p* = 0.02). The endpoint SOS/VOD-related mortality was influenced by allowing DF to treat any emergent SOS/VOD in the control group. The intriguing idea that patients who developed SOS/VOD could benefit from a higher dose of DF was analyzed by Haussmann et al. [31] in a single-center series but was not supported by a randomized phase II dose-finding trial (Study 99–118), which compared 25 mg/kg/day versus 40 mg/kg/day. In this trial, complete response (CR) rates were ~46.7% in the 25 mg/kg arm and ~40.5% in the 40 mg/kg arm. Survival to Day +100 post-HSCT was ~44.0% for the 25 mg/kg vs ~37.8% for the 40 mg/kg group (https://www.accessdata.fda.gov/drugsatfda_docs/nda/2016/208114Orig1s000ClinPharmR.pdf?utm_source=chatgpt.com) [12]. The second trial to assess DF prophylaxis, the Harmony Trial (NCT02851407), involved pediatric and adult patients from 2016 to 2022. The primary outcome was SOS/VOD-free survival at day 30 post-HSCT. Nearly 10 years after the pediatric prevention trial, the estimated incidence was 28%, with a planned sample size of 400 patients. Due to concerns about accurately and consistently diagnosing SOS/VOD, the Harmony Trial employed an independent Endpoint Adjudication Committee (EPAC) that retrospectively evaluated blinded patient data and imaging reports to diagnose SOS/VOD. Unexpectedly, the EPAC identified significantly more cases of SOS/VOD (25% for prophylaxis vs. 21% for best supportive care; BSC) than recorded by the bedside investigators (12% for prophylaxis vs. 16% for BSC). The SOS/VOD-free survival at Day 30 post-HCT was 67% in the DF prophylaxis group compared to 73% in the BSC group (*p* = 0.85). Based on the data, the data safety monitoring committee decided to stop the trial for futility during the interim analysis [32].

The design of the Harmony trial had several notable weaknesses [33], such as including patients with lower risk and overestimating disease incidence [34, 35]. An even bigger challenge than the overestimated incidence was the composite endpoint and the discrepancies between EPAC-based and investigator-based diagnoses, among other issues [33] (trial characteristics of both trials are summarized in Table 2). Nevertheless, the European Medicines Agency (EMA) issued a direct healthcare professional communication (DHPC; https://www.ema.europa.eu/en/medicines/dhpc/defitelio-defibrotide-do-not-use-prophylaxis-veno-occlusive-disease-vod-after-post-hematopoietic) arguing against the use of DF for prophylaxis.

**Table 2:** Trial characteristics of the Pediatric Prevention Trial and the Harmony Trial.

Trial Name	Pediatric Prevention Trial		Harmony Trial				
**Trial number**	NCT00272948		NCT02851407				
**Trial design**	Phase 3, open-label, randomized study		Phase 3, open-label, randomized study				
**Primary outcome parameter**	SOS/VOD incidence by Day 30 post-HSCT		SOS/VOD-free survival by Day 30 post-HSCT				
**Conducted between**	January 2006 and January 2009		August 2016 and March 2022				
**Planned sample size**	290 high-risk pediatric patients (later amended to 360 patients)		400 adult and pediatric patients (trial was stopped with 372 patients)				
**Sample size calculation**	25% with a reduction to 12.5%		28% with a reduction to 16%				
**Assessment of SOS/VOD**	Investigator		Endpoint Adjudication Committee (EPAC)				
**Inclusion criteria**	• 2nd myeloablative HSCT • Allogeneic HSCT for leukemia > 2nd relapse • Pre-existing Liver disease • Prior Abdominal irradiation • Prior treatment with Gemtuzumab • Busulfan/Melphalan Conditioning • Osteopetrosis • Familiar macrophage activating syndrome • Adrenoleukodystrophy (ALD)		• Myeloablative conditioning (MAC) with ○ ≥2 alkylating agents ○ TBI and ≥1 alkylating agent • ≥1 of the following criteria: ○ ≥1 hepatic-related EBMT risk factor1 at screening ○ Advanced-stage neuroblastoma requiring MAC • Osteopetrosis and undergoing MAC • Primary immunodeficiency syndromes • Prior treatment with an OMAB • Class III, high-risk TDT				
**Outcome**	Incidence of SOS/VOD						

	Control Arm	Experimental Arm	Control Arm	Experimental Arm		Control Arm	
				EPAC	Investigator	EPAC	Investigator
	20%	12%	20%	25%	12%	21%	16%

## Defibrotide prophylaxis recommendations

The successful introduction of the pEBMT criteria may raise the question of whether DF prophylaxis is redundant to bridge the gap between the onset of SOS/VOD and preemptive intervention. However, the implications of the DHPC not recommending DF prophylaxis, based on a trial primarily involving lower-risk pediatric and adult patients, could expose some of the highest-risk individuals, such as high-risk infants (who are among the most vulnerable populations undergoing HSCT), to an SOS/VOD-related threat that carries significant morbidity and mortality. It is important to recognize that morbidity, rather than the incidence of SOS/VOD, is another key outcome measure. This is reflected by factors such as the length of hospitalization, the consumption of limited human and medical resources—including intensive care units, extended supportive care, and the need for risky interventions like ventilation and peritoneal drainage—as well as the use of blood and coagulation products. The importance of timely intervention was demonstrated by Szmit et al. [22], who showed that pEBMT criteria, compared to Seattle criteria, significantly influenced these factors. The pediatric prevention trial previously indicated a notable reduction in SOS/VOD-related renal morbidity [8], and Rahim et al. found that patients with SOS/VOD who did not receive prophylactic DF experienced a higher severity at disease onset (very severe 80.9% vs. 66.7%, *p* = 0.592) and a higher rate of maximum severity (very severe 89.4% vs. 83.3%, *p* = 0.532) compared to those who received prophylactic DF [36].

SOS/VOD incidence is linked to significant mortality, independent of the grade of severity. This correlation is confirmed in several publications. Ragoonanan et al. [21] show a significantly higher OS in patients who did not develop SOS/VOD, independent of severity. Rahim et al analyzed the 1-year survival probability among those who did receive prophylactic DF but still developed SOS/VOD (75% vs. 51.1% alive at 1 year) and demonstrated a significantly higher survival rate compared to patients who did not receive DF prophylaxis [36]. Both publications validate the results of the pediatric prevention trial, where the day 100 OS of children who developed SOS/VOD was almost five times higher (25% versus 6%), regardless of disease severity [8]. In conclusion, it is essential to recognize that incidence per se might not be the most appropriate endpoint for assessing the efficacy of DF. Recovery from SOS/VOD, even in severe cases, can often be achieved due to significant advances in managing life-threatening diseases. However, in times of limited human and economic resources, outcome measures should increasingly focus on morbidity rather than mortality. Still, the difference in OS between patients with or without SOS/VOD remains striking and deeply concerning.

## EBMT guidelines for the prophylaxis of SOS/VOD with defibrotide

The arguments outlined above, along with the significant discrepancies between the two pivotal trials and the extensive positive clinical experiences with DF for prevention, have placed the pediatric transplant community in a difficult position due to the EMA’s DHPC, making access to DF for prophylaxis increasingly challenging for pediatric transplant physicians. This situation has led a group of experts to issue recommendations and develop guidelines aimed at supporting pediatric transplant physicians and their teams in identifying high-risk pediatric patients who could benefit from DF for prophylaxis. Additionally, new evidence regarding risk groups has emerged, justifying a reassessment and inclusion in these guidelines (Table 3). The guidelines are organized into sections that define four risk categories, which will be detailed in the following paragraphs:

1. Diseases at high risk for SOS/VOD
2. Diagnostic criteria
3. Iron overload-related liver fibrosis
4. Therapeutic interventions associated with a high risk for SOS/VOD.

**Table 3:** SOS/VOD scoring system.

High risk conditions	Score	Individual score
• Transfusion-dependent thalassemia (TDT)	**1**	
• Myelodysplastic syndrome MDS/juvenile myelomonocytic leukemia (JMML)	**1**	
• Malignant infantile osteopetrosis	**1**	
• High-risk neuroblastoma (HR-NB)	**1**	
• Hemophagocytic lymphohistiocytosis	**1**	
• Liver disease^a^	**1**	
• Prior VOD/SOS^b^	**2**	
• Age <2 years	**2**	
**Diagnostic criteria**		
• Pediatric EBMT criteria	**0**	
• Seattle or Adult EBMT criteria (> day +21)	**1**	
• Obligatory hyperbilirubinemia >2 mg/dl (Baltimore, and others)	**2**	
**Iron overload-related liver fibrosis**^c^		
**Mild** • LIC > 70 to <99 µmol/g dry weight **or** • LSM ≥ 3–7 kPa **or** • Ishak: 0–2 / Metavir: F0–F1	**0**	
**Moderate** • LIC 99 to 200 µmol/g dry weight **or** • LSM ≥ 7.6–10 kPa **or** • Ishak: 3/Metavir: >F2	**1**	
**Severe** • LIC ≥ 200 µmol/g dry weight **or** • LSM ≥ 10 kPa **or** • Ishak: 4–6 / Metavir: F3–F4	**2**	
**Therapeutic interventions**		
• MAC with BU	**1**	
• MAC with BU & a second Alkylator	**2**	
• Prior HSCT with a myeloablative Conditioning^d^	**1**	
• Ozogamicin-conjugated MAB prior to HSCT	**2**	
• Total body irradiation	**1**	
• Gene editing/gene therapy for TDT with BU as conditioning^e^	**2**	
**Cumulative score:**		
**Defibrotide prophylaxis**	**<3 Not recommended** = **3 Advisable** ≥ **4 Recommended**	

*BU* busulfan, *HSCT* hematopoietic stem cell transplantation, *LIC* liver iron content, *LSM* liver stiffness measurement, *MAB* monoclonal antibody, *MAC* myeloablative conditioning, *TDT* transfusion-dependent thalassemia.

^a^Non-iron-related hepatic pathology, including ‚transaminitis‘ prior to HSCT.

^b^After any form of treatment, including conventional chemotherapy.

^c^Ishak/Metavir score overrule scoring according to LIC and LSM.

^d^For each HSCT, one additional score.

^e^No additional score for TDT.

### Diseases at high risk for SOS/VOD

#### Transfusion-dependent thalassemia (TDT)

TDT is a disease with a high transfusion rate, leading to iron overload and liver fibrosis despite optimal chelation, with SOS/VOD incidences ranging from 10 to 21% [37–39]. Recently, the high SOS/VOD incidences were confirmed in TDT patients who received a transplant of genetically modified stem cells after a single-agent myeloablative conditioning with PK-guided BU-based myeloablation [5, 6](see below).

#### Myelodysplastic syndrome (MDS)/juvenile myelomonocytic leukemia (JMML)

Juvenile myelomonocytic leukemia (JMML) is a rare, aggressive myeloproliferative/myelodysplastic disorder affecting infants and children. The reported incidence of SOS/VOD in MDS/JMML cases is as high as 20%. The most recent series published by Sakashita et al. reported an SOS/VOD incidence of 21,4% [40]. In this series, a retrospective analysis of pharmacokinetic (PK) data from samples collected after the first BU dose in 23 patients—6 with SOS/VOD and 17 without—did not show significant differences between the groups, further emphasizing that PK-guided BU-based myeloablative regimens are not fully protective against SOS/VOD [35].

#### Malignant infantile osteopetrosis (MIOP)

MIOP, a rare hereditary osteosclerosing skeletal disorder, is characterized by excessive bone overgrowth caused by a defect in bone marrow resorption by osteoclasts. This leads to the obliteration of bone marrow spaces and subsequent depletion of the hematopoietic stem cell and progenitor compartments, resulting in serious hematologic complications. The incidence of SOS/VOD has been reported to be as high as 60% [29]. This was confirmed in a prospective randomized trial, which reported incidences of 67% in the control arm and 14% in the DF prophylaxis arm [8]. In both reports, BU was the most used alkylator. Shadur et al. [41] reported on thirty-one patients with MIOP who underwent HSCT with a fludarabine/treosulfan/thiotepa (FTT)-based regimen, noting no SOS/VOD and an OS of 100%. Until further corroborating evidence becomes available, MIOP is considered a high-risk disease regardless of the conditioning regimen.

#### High-risk neuroblastoma (HR-NB)

Neuroblastoma (NB) is the most common extracranial solid tumor in children, and high-risk neuroblastoma (HR-NB) remains one of the most difficult diseases in pediatric cancer. Over half of the patients with HR-NB relapse despite intensive multimodal treatment, with most relapses happening within two years of diagnosis. HR-NB mainly affects very young children [42]. As a result, several high-risk factors, such as age, high-dose chemotherapy, autologous HSCT, and the use of BU, add up, increasing the risk for SOS/VOD significantly. There has been little progress in improving disease-free and overall survival in recent decades, especially in the MYCN-amplified group, so high-dose chemotherapy regimens still largely drive treatment protocols [43]. Therefore, the rate of SOS/VOD in HR-NB patients has remained unchanged over the last 20 years, reported at 24% [44] and most recently at 31%, respectively [45].

#### Congenital macrophage activation syndromes (MAS)

Congenital macrophage activation syndromes (MAS), including familial hemophagocytic lymphohistiocytosis (HLH), Griscelli syndrome, and X-linked lymphoproliferative disease, are rare, aggressive, and life-threatening conditions characterized by excessive immune activation. MAS mainly affects infants from birth to 18 months of age, with allogeneic HSCT being the only curative option. The reported incidence of SOS/VOD has remained unchanged over the decades, consistently reported at 30% [8, 46–48]. In addition to the DF prevention trial in children [8], Felber et al. also reported a SOS/VOD incidence of 35%, but only 20% in those who received DF prophylaxis [48]. Most importantly, all patients survived. Wustrau et al. [49] published a series of 60 patients transplanted for HLH with an FTT-based myeloablative conditioning, reporting a SOS/VOD incidence of comparably low 10%. Like other diseases, a combination of individual risk factors—in this case, an allogeneic HSCT in a high-risk disease in infants using BU in myeloablative conditioning—leads to cumulatively high incidences of SOS/VOD. Like MIOP, a change in the alkylator appears to impact the incidence of SOS/VOD significantly. Until further evidence is available, MAS remains a high-risk disease regardless of the conditioning regimen.

#### Preexisting liver diseases and prior SOS/VOD

Preexisting liver diseases, defined as non-iron-related hepatic pathology, including “transaminitis” of unknown origin before HSCT, such as viral hepatitis and prior incidents of SOS/VOD during any treatment, including conventional chemotherapy [1], are well-established conditions that increase the risk of developing SOS/VOD [7, 11, 50, 51]. Strouse et al. [35] developed a risk score for SOS/VOD after HSCT based on patients receiving a first allogeneic HCT between 2008 and 2013 (*N* = 13,097). Independent prognostic factors for SOS/VOD development by day+100 post-HSCT were identified using a multivariate logistic regression model. In this model, positive hepatitis serology was associated with a significantly higher odds ratio (OR).

#### Infants and toddlers

Infants and toddlers, defined as children under 2 years old, are the most vulnerable and largest patient group at a significantly high risk of developing SOS/VOD. Incidences in this population are reported to be as high as 30% [8, 52, 53]. It should be noted that SOS/VOD-high-risk diseases such as HR-NB, MAS, MIOP, and others are most common among infants. The combination of young age, the well-known high-risk diseases, and the considerable variability in BU PKs in this age group can have additive effects that increase the risk of developing SOS/VOD up to 60%, as reported in MIOP [8, 41].

Nevertheless, in the Strouse model [35], younger age was associated with the development of SOS/VOD across all groups. Within the 0–10 years age range, the odds ratio was 1.10, indicating a 10% increased risk for each year younger.

### Diagnostic criteria

The criteria used for diagnosing SOS/VOD significantly affect the incidence, morbidity, and mortality, depending on when the criteria trigger diagnosis and subsequent treatment. Obligatory hyperbilirubinemia (>2 mg/dL) is the hallmark of the Baltimore criteria. However, studies in both adults and children have shown that the overall survival rate at day 100 varies notably between patients with bilirubin levels above and below 2 mg/dL at diagnosis [17], and early intervention is linked to better outcomes [54]. Additionally, anicteric SOS/VOD is common in children, with reported incidences of up to 30%, and up to 15% in adults [8, 17, 55, 56]. Therefore, waiting for bilirubin to rise above 2 mg/dL might delay diagnosis significantly, risking the omission of some anicteric cases. Although the Seattle criteria dropped the requirement for hyperbilirubinemia, recent studies indicate that rRT, the hallmark of the pEBMT criteria, appears to be the most sensitive and earliest indicator of SOS/VOD, diagnosing the condition two to three days earlier than the conventional modified Seattle criteria [20, 23, 24, 57]. Conventional criteria with an apparent delay due to an unusual disease presentation are considered higher risk and are therefore included in the scoring.

### Iron overload-related liver fibrosis

It is well-known that iron overload in the liver causes liver fibrosis and eventually cirrhosis. In addition to primary iron overload in patients with chronic anemia due to increased iron absorption, secondary iron overload can develop in patients who require long-term blood transfusions—most notably in individuals with ß-thalassemia. Despite the use of iron chelation therapy, iron overload and iron-induced liver damage remain unavoidable. The liver’s condition is influenced not only by the level of iron overload but also by epigenetic and environmental factors, which together provoke an inflammatory response. The most important factor is the duration of exposure to toxic iron species (non-transferrin-bound iron and labile plasma iron) [58]. Accordingly, the Pesaro group demonstrated that iron burden per se (serum ferritin, hepatic iron, red cell transfusions) does not determine transplant outcomes.

In contrast, iron-related tissue damage (hepatomegaly, fibrosis/cirrhosis, and poor chelation compliance) does influence transplant outcomes [59, 60]. Therefore, an iron burden exceeding a liver iron content (LIC) of 270 µmol/g dry weight indicates a significant risk of liver damage caused by tissue-reactive iron species and oxidative stress. Conversely, a low iron burden does not necessarily exclude tissue damage [61]. The inflammatory response caused by liver iron varies among individuals. High liver iron content does not necessarily indicate liver damage, and vice versa. Therefore, a liver biopsy before transplant or gene therapy is advisable for personalized risk assessment.

Liver stiffness measurements (LSM) can be obtained using either vibration-controlled transient elastography (VCTE; FibroScan, Echosens, France), point shear wave elastography (p-SWE), or two-dimensional shear wave elastography (2D-SWE). LSM is a highly effective indirect indicator of liver fibrosis and cirrhosis, as recently confirmed in the ELASTOVOD STUDY [62, 63]. Therefore, LSM has been incorporated into the criteria for predicting the SOS/VOD risk score.

In any case, if a high liver iron content is expected due to a presumed high burden (serum ferritin), prolonged exposure (age and number of transfusions), or an abnormal LSM, a liver biopsy is recommended as the gold standard to confirm the extent of liver damage, particularly fibrosis and inflammation. As mentioned above, LSM or LIC do not always correlate with advanced-stage fibrosis or cirrhosis, since the effect of iron-triggered inflammation or fibrosis can vary among individuals.

Eventually, the pre-existing hepatic risk for SOS/VOD was classified using LIC, LSM, and liver biopsy, which, if performed, can even enable “down-staging” if the Ishak/Metavir scores do not match the risk scores of LIC/LSM.

### Therapeutic interventions

#### Busulfan

Busulfan, an alkylating agent frequently used in conditioning regimens for HSCT, has a well-established link to liver sinusoidal endothelial damage, which can lead to SOS/VOD. The risk varies based on dose and is affected by drug exposure (AUC), patient-specific factors such as age, liver function, and prior therapy, as well as concurrent hepatotoxic agents.

Modern studies focus on PK monitoring of busulfan and other strategies to reduce the risk of SOS/VOD. Several studies demonstrate that adding a second alkylator or even non-alkylating agents increases the risk of SOS/VOD [64–68]. The effect of PK monitoring of busulfan on the incidence of SOS/VOD remains a topic of debate [35, 45, 69–71]. In recent years, several changes have been implemented, including high-resolution typing and the substitution of Cy with Flu, which have significantly affected the incidence of VOD/SOS. It is important to note that there is still no standardization or quality control in place. BU dosing ranges from once daily to four times daily, and both application methods are believed to influence SOS/VOD risk. Different algorithms are used to calculate the AUC, which varies accordingly, and the AUC relevant for preventing SOS/VOD is not well defined. This inconsistency is highlighted by Strouse et al., who developed a risk score for SOS/VOD after analyzing over 13,000 patients, where PK-based BU dosing was identified as one of the highest risk factors for VOD/SOS, likely because in half of the patients, dosing is increased to reach the AUC target. Ultimately, Busulfan remains the alkylator with the highest risk of causing SOS/VOD and is therefore included in the scoring system as a significant risk factor, regardless of PK monitoring.

#### Second hematopoietic stem cell transplantation

A second HSCT is a known risk factor for an increased risk of SOS/VOD [7, 11]. While differentiating between reduced-intensity regimens before a second transplant and more recently introduced myeloablative regimens, including treosulfan as the alkylating agent, affects the subsequent risk for SOS/VOD, this remains a topic of debate and lacks conclusive evidence since SOS/VOD was reported for both conditioning options [72]. Partly because treosulfan is regarded as myeloablative only when combined with thiotepa [73], which, as a second alkylator, can pose a risk of VOD/SOS. Other factors, such as the underlying disease and additional considerations, can modify an individual’s risk, so a second or even third transplant must be carefully evaluated when assessing risk.

#### Ozogamicin-conjugated monoclonal antibodies (OMAB)

Ozogamicin-conjugated monoclonal antibodies (OMAB), such as gemtuzumab and inotuzumab, used before HSCT are well-known high-risk factors for SOS/VOD [74–77]. Although this risk can be reduced by increasing the interval between OMAB administration and transplant, lowering the dose, or adjusting the conditioning regimen to promote liver recovery, the effectiveness of these strategies remains unclear. Consequently, these adjustments have not been considered as factors for individual risk assessments or scoring. Similarly, the differences between gemtuzumab and inotuzumab regarding severity are debated. Recent small series involving inotuzumab in adults report an unexpectedly high rate of severe and fatal SOS/VOD [78, 79], which has not been observed with gemtuzumab. Since there is no further evidence to support an alternative approach, the scoring does not differentiate between the two OMABs.

#### Total body irradiation (TBI)

Total body irradiation (TBI) is a well-known risk factor for developing SOS/VOD. In 1987, Jones et al. [14] highlighted the increased risk of SOS/VOD associated with TBI in the influential paper that introduced the criteria later called “Baltimore criteria”. This risk persists today and is included in many risk classifications, as recently summarized by Mohty et al. [11]. Consequently, TBI was also recognized in both prevention trials as a high-risk factor and is part of current recommendations [8, 32].

#### Gene therapy/gene editing in patients with TDT

Gene therapy using the lentiviral-based gene addition approach to introduce a modified adult hemoglobin (HbA) with a T87Q amino acid substitution (HbAT87Q) in the beta chain has proven to be a highly effective treatment for patients with TDT, achieving transfusion independence in 91% of trial participants [6]. Similarly, the non-viral, ex vivo gene editing approach employing CRISPR/Cas9 to knockout the erythroid-specific enhancer region of BCL11A in CD34+ hematopoietic stem and progenitor cells (HSPCs), reducing erythroid-specific BCL11A expression and reactivating HbF, was very successful, with 94.2% [5] of patients achieving transfusion independence.

Both approaches significantly expand the arsenal of effective treatments for patients with TDT. Still, they come with a caveat, using busulfan in their key licensing trials. Despite careful PK monitoring, the incidences of SOS/VOD reached surprisingly high rates of 12.5% and 9,3% in the gene editing and gene addition trials, respectively [5, 80]. This incidence aligns with existing literature on allogeneic HSCT for TDT, which reports an average incidence of 10% to 21% [38, 39]. However, age-related differences mostly affect adolescents and young adults (AYA) with TDT. This is consistent with recent reports of SOS/VOD incidences in young adults aged 18 to 25 being as high as 17%, compared to only about 4% in patients over 25 [81]. This finding is supported by data from the pediatric prevention trial, where the incidence in adolescent high-risk patients was 20% overall [8]. Accordingly, the incidence rates varied by age in both trials, with patients aged 5–11 years experiencing rates of 12.5% and 0%, and those aged 12–17 years experiencing rates of 20% in the gene editing and 23% in the gene addition trials, respectively. This high incidence is notable because, unlike conventional HSCT, both trials used a single alkylator, busulfan, for myeloablation. Due to this unexpectedly high SOS/VOD incidence, the FDA recommended prophylaxis for SOS/VOD in its US Prescribing Information (USPI) for both approaches. Specifically, the recommendation for the gene addition approach (Lyfgenia^TM^) explicitly states the use of ursodeoxycholic acid or DF.

Consequently, bot curative gene-therapeutic approaches were included in the scoring.

#### Cumulative risk-score

Each risk factor has an individual score, and these scores cumulatively reflect the total risk score for each patient (Table 3). The three categories were established to indicate predictive SOS/VOD incidence rates of more than 15%, between 15% and 10%, and below 10%. Therefore, a cumulative score of “4” and higher indicates a significant risk of developing SOS/VOD, which strongly supports the use of DF prophylaxis, based on a predicted risk of developing SOS/VOD post-transplant of 15% or higher. Similarly, a cumulative score of “3” suggests a potential post-transplant SOS/VOD risk of above 10% and supports the use of DF prophylaxis, while a score of “2” or lower does not warrant the use of SOS/VOD prophylaxis.

## Conclusion

SOS/VOD remains a significant post-transplant complication, particularly with a high incidence among children. This group of experts believes that certain cumulative risk factors, including diseases, conditions, and treatment options, can expose specific pediatric populations to a particularly high risk of SOS/VOD. Therefore, prophylaxis with DF with its proven safety and efficacy is a reasonable approach to reduce its incidence and, more importantly, its impact on morbidity and the severity of the disease course.

These guidelines, with their well-established evidence base, are considered a living document that requires ongoing verification to refine and improve patient outcomes over the long term as additional data emerge.

## Data Availability

The content of this manuscript is guidelines published by the Pediatric Diseases Working Party of the EBMT, based on expert opinion supported by published data as indicated in the References list of the manuscript. All data backing the study’s findings are publicly accessible.
